# Improved Monitoring of Low-Level Transcription in *Escherichia coli* by a β-Galactosidase α-Complementation System

**DOI:** 10.3389/fmicb.2019.01454

**Published:** 2019-06-26

**Authors:** Yan Guo, Chang-Ye Hui, Lisa Liu, Hao-Qu Zheng, Hong-Min Wu

**Affiliations:** ^1^Department of Science & Education, Shenzhen Prevention and Treatment Center for Occupational Diseases, Shenzhen, China; ^2^Department of Pathology & Toxicology, Shenzhen Prevention and Treatment Center for Occupational Diseases, Shenzhen, China; ^3^Institute of Translational Medicine, Shenzhen Second People’s Hospital, Shenzhen, China

**Keywords:** transcriptional signal, *E. coli*, fluorescence, β-galactosidase, α-complementation

## Abstract

Genetically encoded reporter proteins are important and widely used tools for the identification and capture of a promoter, tracking the dynamic behavior of transcription, and the quantification of promoter activity. The sensitivity of the reporter gene is a critical factor for an ideal reporter system because weak transcriptional signal has usually failed to be detected using classical reporters. In this study, we present a novel reporter system for improved monitoring of transcription in *E. coli* based on β-galactosidase α-complementation. In this reporter system, the β-galactosidase activity resulting from the assembly of a reporter lacZα and an existing α-acceptor in advance serves as a measure of transcriptional activity *in vivo*. To validate the potential of the lacZα-derived reporter system, a series of artificial operons were constructed, and the moderately strong *lac* promoter, *ara* promoter, and weak *pbr* promoter were chosen as the detection promoters. The response profiles of lacZα was similar to that of wild type lacZ in artificial *lac* operons. Due to its small size and efficient expression profile, the detection sensitivity of a lacZα-derived reporter system was significantly higher than that of the traditional full-length β-galactosidase and the fluorescent protein mCherry reporter system in artificial *ara* operons. As expected, the response sensitivity of the lacZα-derived reporter system was also demonstrated to be significantly higher than that of the β-galactosidase and mCherry reporter systems in lead-sensitive artificial *pbr* operons. The lacZα-derived reporter system may prove to be a valuable tool for detecting promoter activity, especially low-level transcription *in vivo*.

## Introduction

Bacteria dedicate an enormous amount of effort to regulate gene expression in response to environmental or physiological factors. Promoters control the transcription of all genes, and promoter activities are frequently regulated by changes in environmental or physiological conditions (Cases et al., 2003; Martinez-Antonio and Collado-Vides, 2003). Transcriptional fusions have been demonstrated to be important tools in studying gene expression and gene regulation in living organisms (Miller et al., 2000; Flores-Sandoval et al., 2015). Monitoring promoter activity is greatly facilitated by using reporter genes. Many genetically encoded reporter proteins have been successfully used for quantitative, non-disruptive monitoring of transcription in a living organism (Magrisso et al., 2008; Carter et al., 2014; Hui et al., 2019).

The widely used enzymatic reporters that are used to characterize transcription exhibit many strengths. The great advantage of an enzymatic approach is its relatively high sensitivity because even a low-level production of enzyme can, over time, catalytically hydrolyze enough substrate molecules to produce a detectable signal (Kawano et al., 2005). In addition, colorimetric detection of enzymatic activity with the naked eye using convenient and inexpensive plate assays is usually possible (Duttweiler, 1996; Fuxman Bass et al., 2016). Classical enzymatic reporters, such as β-galactosidase, hydrolyze an externally supplied substrate and yield a detectable product. β-galactosidase has been widely used as a reporter both *in vivo* (Asanuma et al., 2015; Gu et al., 2016) and *in vitro* (Seeber and Boothroyd, 1996; Kita et al., 2008). β-galactosidase has a molecular weight of 540 kDa, and previous studies suggest that transcripts from many potential promoters are not detected because of a low expression level of high molecular weight reporter proteins (Kawano et al., 2005). Thus, there is an urgent need to develop a small-molecule reporter protein or peptide for enhanced detection sensitivity.

The intracistronic α-complementation, a property of the *lacZ* gene, has been well characterized and adapted in many studies including the blue-white screening of recombinant clones, live-cell dynamic sensing of protein–protein interactions, and so on (Ahmad et al., 2012; Mogalisetti and Walt, 2015). The lacZM15 is a β-galactosidase deletion mutant lacking N-terminal residues 11–41. The production of lacZM15 can be induced by the analogs of lactose in specific laboratory strains of *Escherichia coli*, such as Top10, DH5α, and JM109 (Jacobson et al., 1994). The resultant protein lacZM15, also known as an α-acceptor, is dimeric and inactive. The mechanism of α-complementation most likely involves an initial binding of two α-donor peptides to the lacZM15 dimer, followed by the slow and essentially irreversible formation of active β-galactosidase with a native-like tetramer (Mogalisetti and Walt, 2015).

Many α-donor peptides with varying lengths have been investigated for α-complementation, and a peptide containing less than 37 residues was required for α-donor activity (Nishiyama et al., 2015). Excessive redundant sequences may lead to lower expression levels in bacterial cells. However, a short peptide chain may lead to lower stability *in vivo*. It was found that short α-donor peptides were generally degraded very rapidly, while longer α-donor peptides were more stable (Zabin, 1982; Jacobson et al., 1994).

Unlike the previous studies, the lacZα gene was chosen as a reporter gene rather than a full-length β-galactosidase gene in this study. Inductive production of α-donor lacZα peptide encoded by reporter vectors and α-acceptor lacZM15 encoded in the host genome can be conveniently assembled into an active enzyme in the host cell. LacZα production-inducing β-galactosidase activity can finally be detected in traditional enzymatic assays. Polypeptide expression always imposes a lower energy consumption on host cells than the full-length protein does. Due to the efficient expression profile resulting from its small size, the detection sensitivity of a lacZα-derived system was expectedly increased. The improved detection sensitivity makes the lacZα-derived reporter system a potential tool in the analysis of weak promoter activity.

## Materials and Methods

### Bacterial Strains and Vectors

The *E. coli* strains and plasmids used in this study are listed in Table 1. Chemically competent *E. coli* Top 10 was used as a host for the cloning and propagation of plasmids. *E. coli* Top 10 and DH5α were used for induced expression of plasmid-coded red fluorescence protein mCherry, lacZ, and lacZα peptide. *E. coli* was grown in Lysogeny Broth (LB) (1% w/v tryptone, 0.5% w/v yeast extract, and 1% w/v NaCl) supplemented with 1% w/v glucose and 50 μg/mL ampicillin (Amp), as necessary (Bertani, 2004). All molecular biology reagents were obtained from TaKaRa (Dalian, China). 5-bromo-4-chloro-3-indolyl-β-D-galactoside (X-gal), Isopropyl-β-D-thiogalactopyranoside (IPTG), 2-Nitrophenyl-β-D-galactopyranoside (ONPG), L-arabinose, antibiotics, and lysozyme were purchased from Sangon Biotech (Shanghai, China). Tryptone and yeast extract were obtained from OXOID (Basingstoke, United Kingdom). All chemicals were purchased from Sigma-Aldrich (Indianapolis, United States). All oligonucleotides and some fusion genes were synthesized by Sangon Biotech (Shanghai, China).

**TABLE 1:** Bacterial strains and plasmids used in this study.

**Strains/plasmids**	**Genotypes or description**	**Source**
*E. coli* Top10	F^–^, Φ80*lac*ZΔM15, Δ*lac*X74, *rec*A1	Invitrogen
*E. coli* DH5α	F^–^, Φ80*lac*ZΔM15 Δ(*lac*ZYA-*arg*F), U169, *rec*A1	Invitrogen
pBR322	Amp^R^, Tet^R^, *ori pMB1*, commonly used *E. coli* cloning vector	Novagen
pUCm-T	TA cloning	Sangon
pBAD	Amp^R^, *ori pBR322*, containing *ara*BAD promoter	Thermo Fisher Scientific
pT-RFP	pUCm-T carrying *mCherry*	Hui et al.,2018c
pPlac-lacZα	Amp^R^, *ori pMB1*, pBR322 derivative with lacZα peptide expressing under *lac* promoter	This study
pPlac-lacZ	pBR322 derivative with β-galactosidase expressing under *lac* promoter	This study
pPlac-RFP	pBR322 derivative with mCherry protein expressing under *lac* promoter	This study
pPara-lacZα	Amp^R^, *ori pMB1*, pBR322 derivative with lacZα peptide expressing under *ara*BAD promoter	This study
pPara-lacZ	pBR322 derivative with β-galactosidase expressing under *ara*BAD promoter	This study
pPara-RFP	pBR322 derivative with mCherry protein expressing under *ara*BAD promoter	This study
pT-Ppbr	pUCm-T carrying *pbrR* and divergent *pbr* promoter region	This study
pPpbr-RFP	pPlac-RFP derivative with *pbrR* and *pbr* promoter preceding *mCherry*, a single RFP reporter system	This study
pPpbr-lacZα	pPlac-lacZα derivative with *pbrR* and *pbr* promoter preceding *lacZα*, a single lacZα reporter system	This study
pPpbr-lacZ	pPlac-lacZα derivative with *pbrR* and *pbr* promoter preceding *lacZ*, a single lacZ reporter system	This study

*The cloning/expression regions of recombinant plasmids used in this study are shown in Supplementary Figure S1.*

### Construction of the *lac* Promoter Reporter Vectors

For cloning purposes, all PCR reactions were carried out for 25 cycles of 2 min at 95°C, 1 min at 50–60°C, and 1–2 min at 72°C. All primers used in this study are listed in Table 2.

**TABLE 2:** Oligonucleotides used in this study.

**Name**	**Sequence 5′–3′**
F-Plac	CACACCGCATATGATTAATGCAGCTGGC
R-Plac-RFP	CTCGCCTTTAGAGACCATAGCTGTTTCCTG
F-Plac-RFP	CAGGAAACAGCTATGGTCTCTAAAGGCGAG
R-RFP-Ter	CTGATTTAATCTGTATTATTTGTACAGTTCG
F-RFP-Ter	CGAACTGTACAAATAATACAGATTAAATCAG
R-Ter	GGAATTCAAGGCCCAGTC
F-Para	CACACCGCATATG TTATGACAACTTGACGG
R-Para	ACGCGTCGAC AGCCCAAAAAAACG
F-RFP	ACGCGTCGAC AGGAGGAATTAACC ATGGTCTCTAAAGGC
F-lacZα	ACGCGTCGAC AGGAGGAATTAACC ATGACCATGATTACGG
F-Ppbr	CACACCGCATATGTTACCCAGATG
R-Ppbr-RFP	CCTTTAGAGACCATGAGTAACTCC
F-Ppbr-RFP	GGAGTTACTCATGGTCTCTAAAGG
R-Ppbr-lacZα	CCGTAATCATGGTCATGAGTAACTCC
F-Ppbr-lacZα	GGAGTTACTCATGACCATGATTACGG
R-Ppbr	ACGCGTCGACGAGTAACTCCTG

A cassette containing the *lac* promoter, a lacZα open reading frame (ORF), and *rrn*B termination region was synthesized by Sangon Biotech (Shanghai, China). The synthesized fragment was then cloned as a *Nde*I/*Eco*RI fragment into pBR322, previously digested with the same enzymes. The resulting plasmid was named pPlac-lacZα. Another fusion cassette containing the *lac* promoter, a *Sal*I restriction enzyme site, a full-length β-galactosidase lacZ ORF, and *rrn*B terminator was also synthesized. The resulting *Plac*-*lacZ-rrn*B terminator gene fusion cassette was then digested and inserted in pBR322 via *Nde*I and *Eco*RI sites, generating Plac-lacZ.

The pPlac-RFP was constructed through a PCR overlap extension method (Miller et al., 2000). First, the *lac* promoter region was amplified from pPlac-lacZα using primers F-Plac and R-Plac-RFP, and *mCherry* was amplified from pT-RFP using primers F-Plac-RFP and R-RFP-Ter. Second, both of the amplified products from the above reactions were added to a second PCR reaction using primers F-Plac and R-RFP-Ter, and the resulting fragment was the *Plac*-*mCherry* cassette. Finally, the *rrn*B terminator was amplified from pPlac-lacZα using primers F-RFP-Ter and R-Ter. Then the amplified product and the *Plac*-*mCherry* cassette were added to a second PCR reaction using primers F-Plac and R-Ter. The resulting *Plac*-*mCherry*-*rrn*B terminator gene fusion cassette and pBR322 were then digested with *Nde*I and *Eco*RI, and ligated together to generate the plasmid pPlac-RFP.

### Construction of the *ara* Promoter Reporter Vectors

The cassette containing the sequence encoding araC and an *ara* promoter region was amplified from pBAD vector using primers F-Para and R-Para. This fragment was then cloned as a *Nde*I and *Sal*I fragment into pPlac-lacZ, previously digested with the same enzymes. The resulting plasmid was named pPara-lacZ. The cassette containing the gene of mCherry and *rrn*B terminator region was amplified from pPlac-RFP using primers F-RFP and R-Ter. The amplified fragment was then cloned as a *Sal*I and *EcoR*I fragment into pPara-lacZ to generate the plasmid pPara-RFP. The cassette containing the gene of lacZα and *rrn*B terminator region was amplified from pPlac-lacZα using primers F-lacZα and R-Ter. The resulting PCR fragment was digested and inserted into pPara-lacZ via *Sal*I and *EcoR*I sites, generating pPara-lacZα.

**FIGURE 1:**
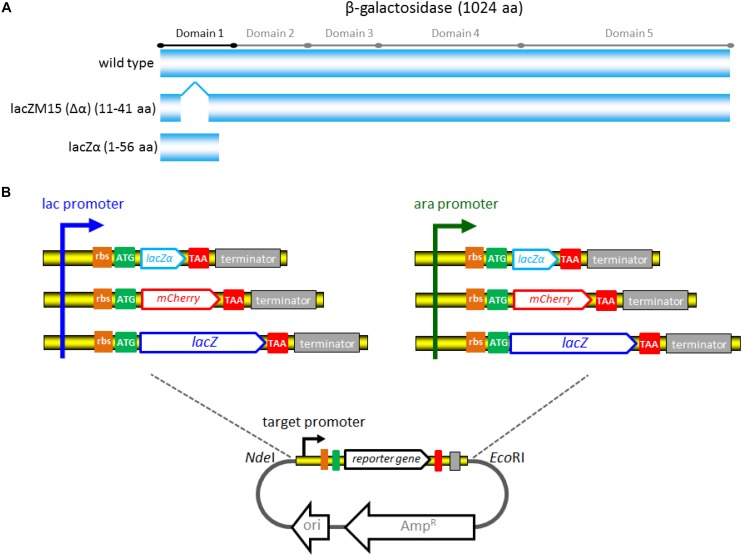
The strategy used for constructing a series of *lac* promoter and *ara* promoter reporter vectors. **(A)** Schematic representation of wild-type β-galactosidase, lacZM15, and lacZα polypeptides. Five distinct domains of the β-galactosidase monomer (1024 aa) was marked. LacZM15 is a deletion mutant (residues 11–41) that maps in the α region. The preferred α-donor peptide lacZα involved in this study contains wild type residues 1–56. α-complemented active β-galactosidase assembled from lacZM15 and lacZα contains two sets of overlapping sequences, which are segments 1–10 and 42–56. **(B)** Assembly strategy for the *lac* promoter and *ara* promoter reporter vectors used in the study. Assembly of the desired target promoter, ribosome binding site, a reporter gene (including the encoding sequence for lacZα, mCherry, or full-length lacZ), and *rrn*B terminator via the genetic methods to construct the reporter cassette. The resulting fusion fragments are then inserted into pBR322 via *Nde*I and *Eco*RI sites.

The strategy used for detection of *lac* and *ara* promoter activity with different reporter systems is summarized in Figure 1. A 2063 bp *Nde*I-*Eco*RI fragment of plasmid pBR322, which only retains the origin of replication of pMB1, and the gene *bla* encoding the ampicillin resistance (Amp^R^) protein, was chosen to be the cloning vector backbone. All promoter reporter vectors share a common plasmid pBR322 backbone, but contain different promoter reporter cassettes. The target promoter, the ribosome binding site (RBS), a reporter encoding sequence and the terminator (derived from *E. coli rrnB* operon) (Brosius et al., 1981) were first fused. The resultant fusion cassette and the clone vector backbone were then assembled with restriction enzymes *Nde*I and *Eco*RI to create the reporter vectors.

### Construction of the *pbr* Promoter Reporter Vectors

The *pbr* operon, originating from *Cupriavidus metallidurans* strain CH34, is a unique lead resistance operon (Borremans et al., 2001; Vandamme and Coenye, 2004; Hui et al., 2018a). Based on the natural *pbr* operon, the lead bacterial biosensors have been successfully developed, and the genetic elements of the lead biosensor constructs consist of the transcriptional factor PbrR gene, together with a divergent *pbr* promoter, and a promoterless reporter fluorescent protein gene (Wei et al., 2014; Bereza-Malcolm et al., 2016). To test the detection sensitivity of *pbr* promoter activity, pPpbr-RFP, pPpbr-lacZα, and pPpbr-lacZ were constructed in this study.

The cassette containing the sequence encoding the transcriptor PbrR and the bidirectional *pbr* promoter region was synthesized by Sangon Biotech (Shanghai, China), and the synthesized fragment (520 bp) was cloned into pUCm-T to generate pT-Ppbr. The lacZα and mCherry *pbr* promoter reporter cassettes were constructed as follows: A lacZα reporter element and a mCherry reporter element were amplified from pPlac-lacZα, and pPlac-RFP, respectively, and fused to the genetic element containing *pbrR* and *pbr* promoter by a PCR overlap extension method. Briefly, a lacZα reporter element was amplified with primers F-Ppbr-lacZα and R-Ter, and a mCherry reporter element was amplified with primers F-Ppbr-RFP and R-Ter. The genetic element containing *pbrR* and *pbr* promoter was amplified from pT-Ppbr using primers F-Ppbr and either R-Ppbr-lacZα or R-Ppbr-RFP. Both of the amplified products from pPlac-lacZα and pT-Ppbr (using primer R-Ppbr-lacZα) were added to a second PCR reaction containing primers F-Ppbr and R-Ter to generate the Ppbr-lacZα gene fusion cassette. Similarly, the second round of PCR with primers F-Ppbr and R-Ter was performed after mixing the amplified product from pT-Ppbr (using primer R-Ppbr-RFP) with the amplified product from pPlac-RFP to generate the Ppbr-RFP gene fusion cassette. The two Ppbr reporter cassettes above and pBR322 were finally digested with *Nde*I and *Eco*RI, and ligated together to generate pPpbr-lacZα, and pPpbr-RFP, respectively. The cassette containing the sequence encoding PbrR and a *pbr* promoter region was amplified from the pT-Ppbr vector using primers F-Ppbr and R-Ppbr. This fragment was then cloned as a *Nde*I and *Sal*I fragment into pPlac-lacZ, previously digested with the same enzymes. The resulting plasmid was named pPpbr-lacZ.

**FIGURE 2:**
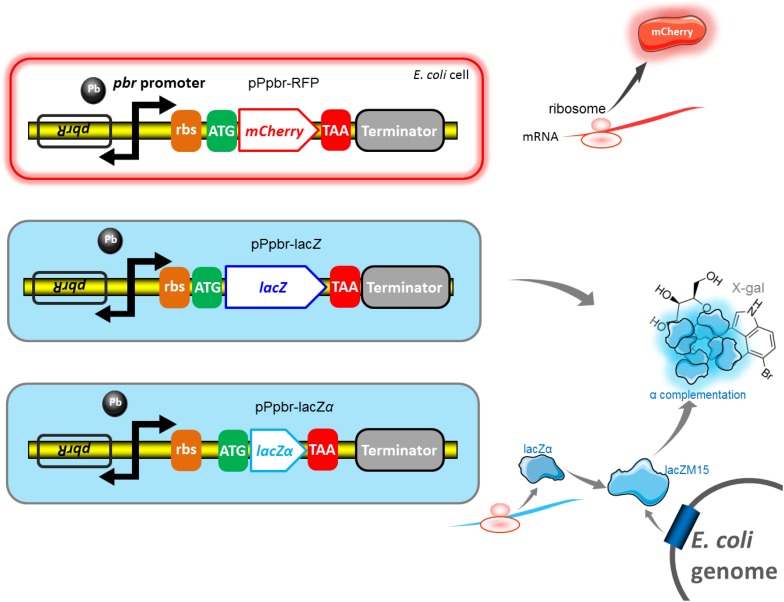
Genetic organization of the *pbr* promoter reporter systems. The lead(II) inducible mCherry reporter expression, full-length lacZ reporter expression, and lacZα reporter expression are achieved in *E. coli* Top10 harboring pPpbr-RFP, pPpbr-lacZ, and pPpbr-lacZα, respectively. Based on a well-known monocistronic reporter cassette, fluorescent signal and enzymatic activity are detected under Pb(II) induction. LacZα and lacZM15 are separately synthesized under the control of target *pbr* promoter driven by Pb(II), and host *lac* promoter driven by IPTG. After the active β-galactosidase with a native-like tetramer is finally assembled *in vivo*, a standard chromogenic substrate method can then be used for qualitative and quantitative determination of β-galactosidase activity.

The strategy and potential mechanism involved in three reporter systems are shown in Figure 2. β-galactosidase α-complementation can be achieved by assembling the lead(II) inductive lacZα derived from the plasmid with the IPTG inductive lacZM15 derived from the host cell genome.

### Reporter Genes Expression

*Escherichia coli* hosts were transformed with recombinant vectors using a CaCl_2_-mediated transformation method (Hui et al., 2018b). The transformed *E. coli* cells were spread onto LB agar plates containing 50 μg/mL ampicillin, and cultured overnight at 37°C. A single colony picked from an agar plate was used to inoculate 3 mL of LB medium supplemented with 50 μg/mL ampicillin in a 15 mL Bio-Reaction tube (Jet, Guangzhou, China), and cultured for 12 h in a 37°C shaking incubator at 200 rpm.

For IPTG-induced reporter genes expression, recombinant *E. coli* harboring the *lac* promoter reporter vectors were cultured in LB medium for 12 h, and diluted to an OD_600_ of 0.01 in 12 mL fresh LB medium supplemented with 1% glucose and 50 μg/mL ampicillin in a 50 mL Bio-Reaction tube. The culture was grown at 37°C for 3 h with rotation at 220 rpm, and the bacteria reached log phase with an optical density 0.4 at 600 nm. The cultures were then induced with 0–1.0 mM IPTG and incubated at 37°C with shaking at 220 rpm. Induced cultures were sampled and evaluated for the expression of reporter genes at regular intervals.

For arabinose-induced reporter genes expression, recombinant *E. coli* harboring the *ara* promoter reporter vectors were cultured in LB medium for 12 h, and diluted to an OD_600_ of 0.01 in 12 mL fresh LB medium supplemented with 50 μg/mL ampicillin plus 0.1 mM IPTG in a 50 mL Bio-Reaction tube. The cultures were grown at 37°C for 3 h with rotation at 220 rpm, and then induced with varying concentrations of arabinose. Induced cultures were incubated at 37°C with shaking at 220 rpm, and tested for the expression of reporter genes at regular intervals.

For Pb(II)-induced reporter genes expression, recombinant *E. coli* harboring the *pbr* promoter reporter vectors were cultured in LB medium for 12 h, and diluted to an OD_600_ of 0.01 in 12 mL fresh LB medium supplemented with 50 μg/mL ampicillin in a 50 mL Bio-Reaction tube. The cultures were grown at 37°C for 3 h with rotation at 220 rpm, and then induced with varying concentrations of Pb(II) plus 0.1 mM IPTG. Induced cultures were incubated for 4 h at 37°C with shaking at 220 rpm, sampled and subjected to reporter gene assays.

### Enzymatic Assay of β-Galactosidase Using *E. coli* Cell Lysate

An enzymatic assay of β-galactosidase activity using cell lysate was performed and modified as previously described (Tomizawa et al., 2016). In brief, *E. coli* cells were collected, washed twice with 50 mM phosphate buffer saline (PBS, pH 7.0), and resuspended in 50 mM PBS. After the OD_600_ of the resuspended cells was measured, an equal volume of assay solution (1 mg/mL lysozyme, 2 mM X-gal, 200 mM NaCl, 50 mM PBS) was added, vortexed for 2 min, and incubated at 37°C for 30 min. The supernatant was obtained by centrifuging at 3500 rpm for 5 min, and then the absorbance was measured at 630 nm using an iMark microplate reader (Bio-Rad, United States). The background value was obtained from the control assays with uninduced cell lysate, and the optical density at 630 nm was then normalized by the OD_600_ value of cell suspensions.

### Fluorescence Quantitative Analysis

The fluorescence intensity of mCherry produced in *E. coli* was measured as previously described (Hui et al., 2018c). Briefly, *E. coli* cells were collected, washed, and resuspended in 50 mM PBS. A 3-mL aliquot of *E. coli* suspension or diluent was added to 1-cm low fluorescence background quartz cuvette. To test the fluorescence intensity of induced mCherry, the excitation wavelength was set at 587 nm and the intensity of emitted fluorescence of mCherry at 610 nm was recorded with Lumina fluorescence spectrometer (Thermo Fisher Scientific, United States). The fluorescence intensity was normalized by dividing the fluorescence intensity at the emission wavelength 610 nm by the OD_600_ value of the same sample. The background value was obtained from the control assays with uninduced cell suspensions.

### Plate Induction Assay

Single colonies were picked from each construct and inoculated in 3 mL LB medium with 50 μg/mL ampicillin at 37°C for 6 h. The precultured recombinant *E. coli* was plated on LB plate containing optimal concentration of inducer for assay, or no inducer for the control, cultured at 37°C for 18 h. The LB agar was supplemented with 0.6 mM IPTG to induce the transcription of *lac* promoter, 0.002% arabinose plus 0.1 mM IPTG to induce the transcription of *ara* promoter, and 20 μM Pb(II) plus 0.1 mM IPTG to induce the transcription of *pbr* promoter. To examine the expression of reporter lacZα and LacZ, extra substrate 0.4% X-gal or 0.4% ONPG was also added to LB agar.

## Results and Discussion

### Detection of *lac* Promoter Activity With lacZα Production-Inducing β-Galactosidase

In order to compare the transcriptional and translational levels of the novel lacZ α-complementation reporter system, the full-length β-galactosidase, and the commonly used fluorescent protein reporter system, a monocistronic lacZα, lacZ, and mCherry reporter vectors based on an artificial *lac* operon were assembled, and named pPlac-lacZα, pPlac-lacZ, and pPlac-RFP, respectively.

**FIGURE 3:**
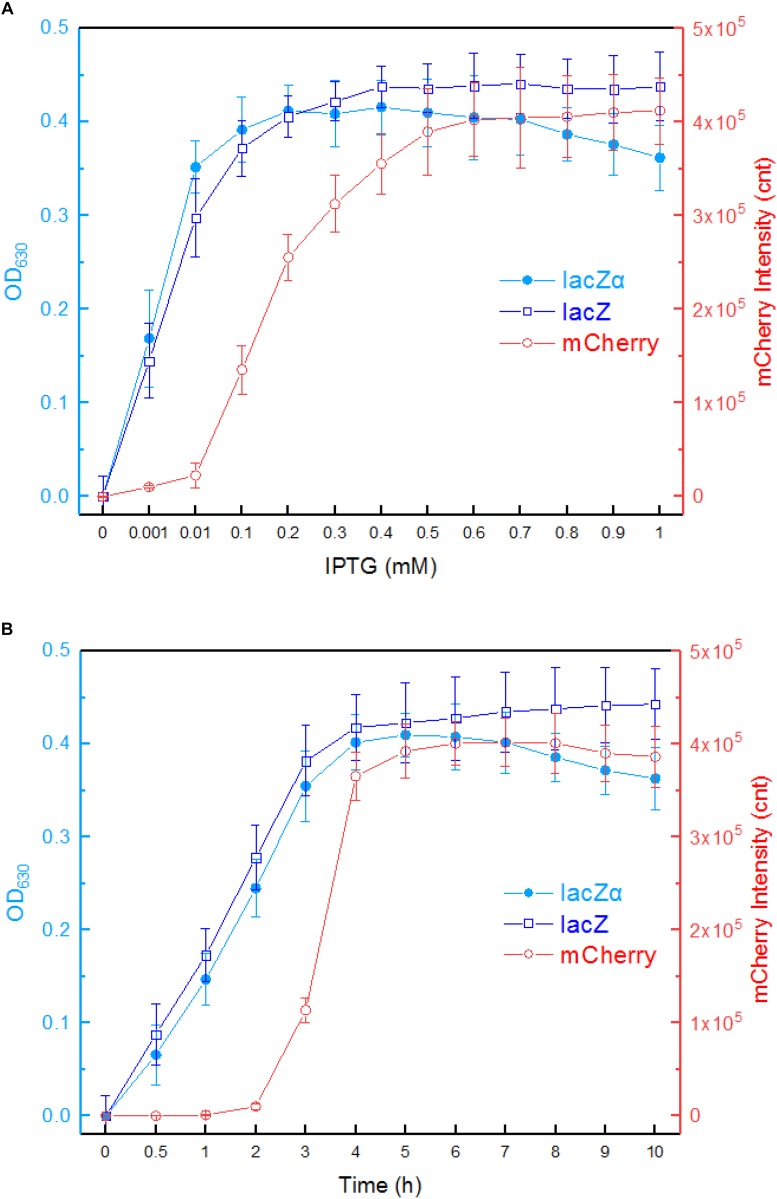
Comparison of *lac* promoter activities in response to IPTG in lacZα, lacZ, and mCherry reporter systems. **(A)** Response curves of Top10/pPlac-lacZα, Top10/pPlac-lacZ, and Top10/pPlac-RFP to different concentrations of IPTG. Recombinant *E. coli* was first induced with 0–1 mM IPTG at 37°C for 8 h. Then, β-galactosidase activity and mCherry fluorescent signal of induced cultures were determined. **(B)** Time courses of reporter signals in recombinant Top10/pPlac-lacZα, Top10/pPlac-lacZ, and Top10/pPlac-RFP treated with 0.6 mM IPTG. Recombinant *E. coli* was induced with 0.6 mM IPTG, and β-galactosidase activity and mCherry fluorescent signal of induced cultures were determined at regular time intervals. The data are representative of three independent experiments and expressed as mean ± SEM. The optical density at 630 nm and the mCherry fluorescence intensity were all normalized by the OD_600_ value of the induced culture.

First, we investigated the response profiles of recombinant *E. coli* Top10 harboring pPlac-lacZα, pPlac-lacZ, and pPlac-RFP in response to varying concentrations of IPTG. After bacterial cells in logarithmic growth were exposed to 0–1.0 mM IPTG for 8 h at 37°C, β-galactosidase activity was assayed in Top10/pPlac-lacZα and Top10/pPlac-lacZ cultures, and mCherry fluorescent intensity was determined in Top10/pPlac-RFP cultures. The results are shown in Figure 3A. Top10/pPlac-RFP was found to respond to the lowest concentration of IPTG at 0.1 mM. However, even when the concentration of IPTG reached as low as 0.001 mM IPTG, β-galactosidase activity was still detected in both Top10/pPlac-lacZα and Top10/pPlac-lacZ cultures. With the increase of IPTG concentration, although significantly higher response levels of three reporters were observed, there was no significant difference of β-galactosidase activities in Top10/pPlac-lacZα and Top10/pPlac-lacZ with 0.001–0.2 mM IPTG induction. The highest response levels of Top10/pPlac-lacZα and Top10/pPlac-lacZ were obtained at about 0.2 mM IPTG, and 0.4 mM IPTG, respectively. The highest response level of Top10/pPlac-RFP was obtained at about 0.6 mM IPTG. The preferred concentration of IPTG was finally determined to be 0.6 mM for the following response time assay.

Second, response times of Top10/pPlac-lacZα, Top10/pPlac-lacZ, and Top10/pPlac-RFP were analyzed following exposure to 0.6 mM IPTG. The results are shown in Figure 3B. Cultures were sampled at consecutive time intervals after IPTG exposure. An approximate 3-h delay in the mCherry fluorescent signal was observed in Top10/pPlac-RFP cultures, and the highest fluorescent intensity was obtained at around 6 h. The response times of Top10/pPlac-lacZα and Top10/pPlac-lacZ were as low as 0.5 h. β-galactosidase activity derived from both lacZα and wild type lacZ increased with prolongation of the induction time up to 4 h, at which point the enzyme activity reached a maximum. Then, the enzyme activity of Top10/pPlac-lacZα decreased gradually, and remained at approximately 88% of the maximum at 10 h.

The production of active β-galactosidase depends on the expression of both α-donor lacZα peptide and α-acceptor lacZM15 protein (Mogalisetti and Walt, 2015). The inductive expression profile of lacZα encoded in a reporter vector might be variable in different hosts. To further validate the performance of lacZα reporter system in different hosts, we examined the response levels of Top10/pPlac-lacZα and DH5α/pPlac-lacZα in response to 0.001, 0.01, 0.1, and 1.0 mM IPTG, respectively. IPTG at varying concentrations was added to an exponential phase bacterial culture and incubated for 4 h at 37°C. Interestingly, Top10/pPlac-lacZα was found to produce significantly higher β-galactosidase activity at all IPTG concentrations examined, in comparison to DH5α/pPlac-lacZα (Supplementary Figure S2). It is well known that the expression of recombinant protein is determined by host genetic background, plasmid copy number, inducer concentration, culture conditions, and so on (Hortsch and Weuster-Botz, 2011; Mahalik et al., 2014). *E. coli* Top10 was then chosen as a preferred host for the following tests.

Furthermore, β-galactosidase activity was not elevated above 0.1 mM IPTG induction, and there was no significant difference in the growth curves of Top10/pPlac-lacZα and DH5α/pPlac-lacZα with 0–0.1 mM IPTG induction (data not shown). Thus, 0.1 mM IPTG was used for the induced expression of lacZM15 in the following promoter activity assays.

### A lacZα Reporter System for Improving Detection Sensitivity of *ara* Promoter Activity

To further determine the potential of lacZα reporter system in detecting promoter activity using a promoter other than the *lac* promoter, a monocistronic lacZα, lacZ, and mCherry reporter constructs based on an artificial *ara* operon were assembled.

**FIGURE 4:**
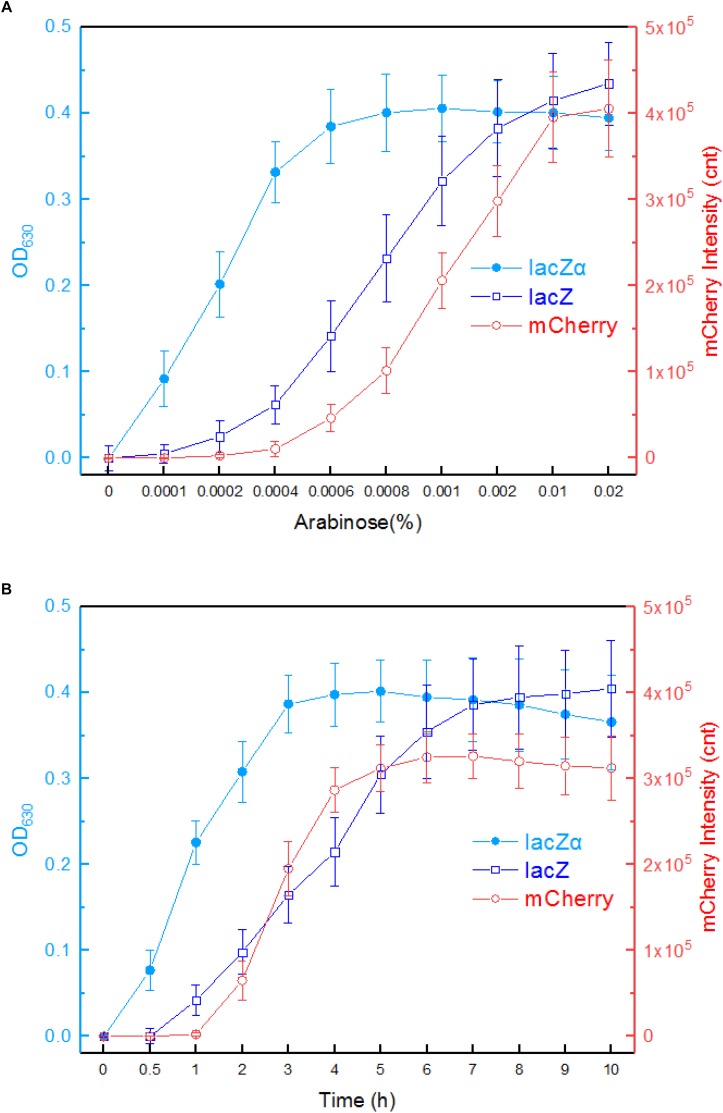
Comparison of the *ara* promoter activities in response to arabinose in lacZα, lacZ, and mCherry reporter systems. **(A)** Response curves of Top10/pPara-lacZα, Top10/pPara-lacZ, and Top10/pPara-RFP to different concentrations of arabinose. Recombinant *E. coli* was induced with 0–0.02% arabinose plus 0.1 mM IPTG at 37°C. Then, β-galactosidase activity and mCherry fluorescent signal of induced cultures were determined at 8 h. **(B)** Time courses of reporter signals in recombinant Top10/pPara-lacZα, Top10/pPara-lacZ, and Top10/pPara-RFP treated with 0.002% arabinose. After recombinant *E. coli* was induced with 0.002% arabinose plus 0.1 mM IPTG, β-galactosidase activity and mCherry fluorescent signal of induced cultures were determined at regular time intervals. The data are representative of three independent experiments, and expressed as mean ± SEM. The optical density at 630 nm and mCherry fluorescence intensity were all normalized by the OD_600_ value of the induced culture.

The response profiles of Top10/pPara-lacZα, Top10/pPara-lacZ, and Top10/pPara-RFP in response to varying concentrations of inducer arabinose was first determined. After recombinant *E. coli* in logarithmic growth were exposed to 0–0.02% arabinose for 8 h at 37°C, β-galactosidase activity and mCherry fluorescent intensity were assayed. As shown in Figure 4A, Top10/pPara-lacZα was demonstrated to respond to as low as 0.0001% arabinose. With the increase of arabinose concentration, significantly increased response of lacZα production-inducing β-galactosidase activity was achieved. The highest response level of Top10/pPara-lacZα was obtained at 0.0006% arabinose. However, Top10/pPara-lacZ and was found to respond to the lowest concentration of arabinose at 0.0002% after 8-h induction. Although the response level of Top10/pPara-lacZ always increased with the increase of arabinose concentration, the response level of full-length lacZ reporter was still significantly lower than that of lacZα reporter with 0.0001–0.002% arabinose induction. Top10/pPara-RFP was found to respond to the lowest concentration of arabinose at 0.0006% after 8-h induction, and the highest response level of Top10/pPara-RFP was obtained at 0.001% arabinose.

Response times of Top10/pPara-lacZα, Top10/pPara-lacZ, and Top10/pPara-RFP were analyzed following induction with 0.002% arabinose. The results are shown in Figure 4B. An approximate 2-h delay in the mCherry fluorescent signal was observed in Top10/pPara-RFP, and the highest fluorescent intensity was obtained at 6 h. An approximate 1-h delay in wild type lacZ activity was observed in Top10/pPara-lacZ, and the lacZ activity increased with prolongation of the induction time up to 10 h. Interestingly, nearly no time delay was observed in lacZα production-inducing β-galactosidase activity. In addition, the response level of lacZα reporter increased with prolongation of the induction time up to 3 h, at which point the enzyme activity had reached a maximum. Importantly, the response level of lacZα reporter was always higher than that of full-length lacZ reporter within a 6-h induction, and the biggest difference between the two groups occurred within a 4-h induction.

The inducible pattern of lacZα reporter is different from a standard lacZ reporter, in which a truncated lacZα is substituted for a wild type lacZ as a reporter. Importantly, the expression of lacZM15 is independent of the inducible expression of lacZα. Through α-complementation, lacZα peptide can be used as a reporter for the target promoter, because α-acceptor lacZM15 is pre-expressed in the host cell in advance. Obviously, induced expression of a small peptide lacZα would be easier and faster in *E. coli* cells than induced expression of a large-sized lacZ.

### Lead(II) Biosensor Using the lacZα Reporter System for Significantly Enhanced Lead(II) Response

**FIGURE 5:**
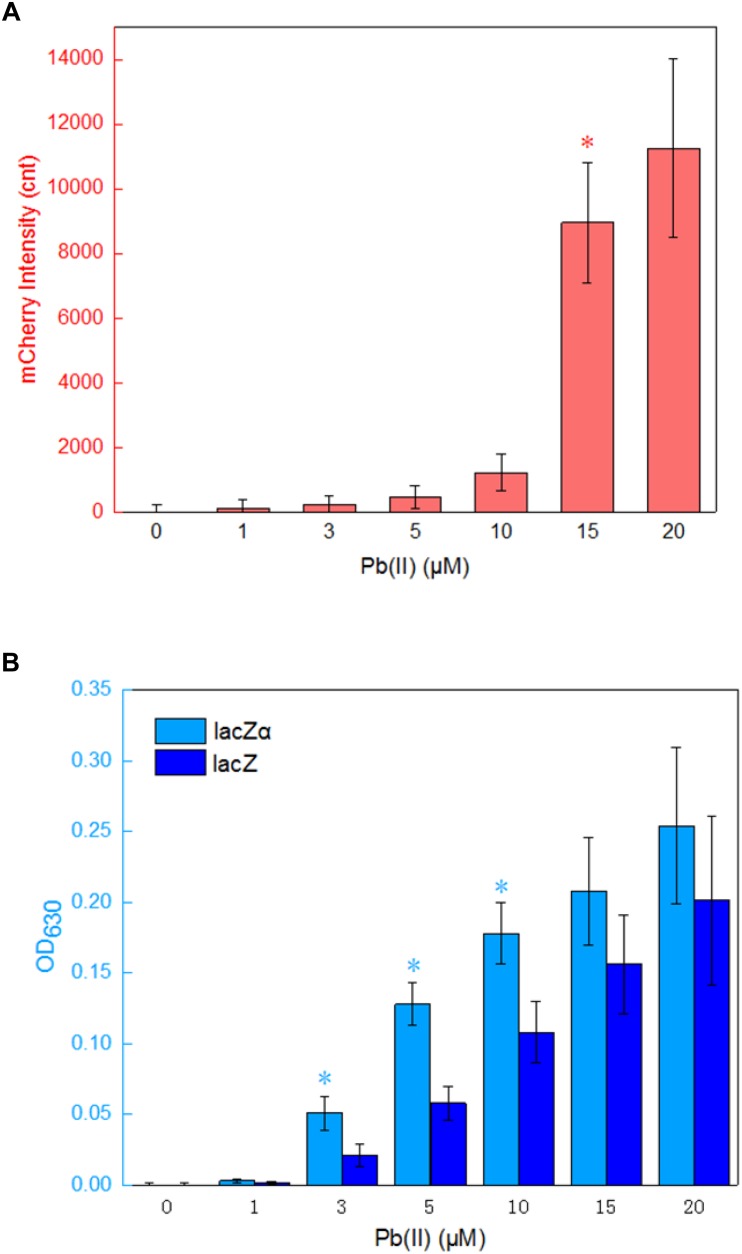
Assay of the *pbr* promoter activities in response to lead(II). **(A)** The response of Top10/pPpbr-RFP to different concentrations of Pb(II) plus 0.1 mM IPTG after 4 h incubation at 37°C. ^*^A significant increase (*t*-test, *P* < 0.05) in the reported signal, in comparison to the same recombinant bacteria with no Pb(II) exposure. **(B)** The response of Top10/pPpbr-lacZα and Top10/pPpbr-lacZ to different concentrations of Pb(II) plus 0.1 mM IPTG after 4 h incubation at 37°C. ^*^A significant increase (*t*-test, *P* < 0.05) between Top10/pPpbr-lacZα and Top10/pPpbr-lacZ. The optical density at 630 nm and mCherry fluorescence intensity were all normalized by the OD_600_ value of the induced culture. The data are representative of three independent experiments, and expressed as mean ± SEM.

The application of lacZα reporter system is limited in lacZM15-producing bacteria, and this is its only disadvantage. When a moderately strong *ara* promoter was placed in front of three reporter cassettes, the results above indicate that the most sensitive response was achieved in a lacZα reporter vector. To examine whether the lacZα-derived report system can be used to detect low-level transcriptional signals from a weak promoter other than *ara* promoter, a 520 bp fragment containing *pbrR* and *pbr* promoter was placed in front of promoterless mCherry, lacZ, and lacZα reporter cassettes. The *pbr* promoter activity was then evaluated by assays of both mCherry and β-galactosidase activity. *E. coli* transformed with Ppbr-RFP, Ppbr-lacZ, and Ppbr-lacZα showed increased signals in response to elevated concentrations of Pb(II). The results are shown in Figure 5.

Recombinant Top10/pPpbr-RFP, Top10/pPpbr-lacZα, and Top10/pPpbr-lacZ in logarithmic growth were exposed to varying concentrations of Pb(II) plus 0.1 mM IPTG for 4 h at 37°C. Top10/pPpbr-RFP, a lead biosensor with a single mCherry reporter system, detected 15 μM Pb(II) (Figure 5A). This result was basically consistent with previous findings (Bereza-Malcolm et al., 2016). However, the lacZα peptide, like full-length lacZ, was demonstrated to be a much more sensitive reporter to detect low transcriptional activity. Both Top10/pPpbr-lacZα and Top10/pPpbr-lacZ were demonstrated to respond to concentrations as low as 3 μM Pb(II) (Figure 5B). It is worth mentioning that the responses from both lacZα and lacZ report systems were associated with Pb(II) exposure in a dose-response manner, and lacZα production-inducing β-galactosidase activity was significant higher than wild type β-galactosidase activity with 3–10 μM Pb(II) induction. However, no significantly higher response from the mCherry reporter system was observed with the increase of Pb(II) in the study. These results suggest that even a weak promoter with very poor transcriptional activity may still be detected with a lacZα-derived reporter system.

### A lacZα-Derived Reporter System Can Be Conveniently Observed in a Plate Assay

**FIGURE 6:**
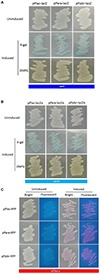
Expression of the three reporters in recombinant *E. coli* Top10. The strains harboring the *lac*, *ara*, and *pbr* promoter reporter systems presented β-galactosidase **(A,B)** or mCherry signal **(C)** when cultured in the presence of 0.06 mM IPTG, 0.002% arabinose plus 0.1 mM IPTG, and 20 mM Pb(II) plus 0.1 mM IPTG, respectively. Extra substrate, 0.4% X-gal or 0.4% ONPG, was added to LB plate for the detection of β-galactosidase activity.

The activity of a target promoter in response to an inducer can be quantified in the liquid media. Moreover, like the expression of mCherry and wild type β-galactosidase, the activation of β-galactosidase in lacZα-derived reporter systems could also be clearly observed with the naked eye in a plate assay (Figure 6). Both X-gal and ONPG could be used as substrates for the chromogenic reaction to indicate the production of active β-galactosidase.

## Conclusion

Enzyme fragment complementation has been used as a selection marker for the rapid detection of recombinant bacteria, protein-protein interaction, high throughput screenings, and so on. In the current study, β-galactosidase α-complementation proves to be a novel tool for transcription monitoring in *E. coli* for the first time. The variable production of lacZα, a small fragment of β-galactosidase, is driven by the detection promoter, and the expression of an inactive lacZ deletion mutant is independently driven by a natural *lac* promoter located in the host genome. The resultant stable heteromeric enzyme complex can be easily detected using a chromogenic substrate, X-gal, which forms an intense blue precipitate when hydrolyzed. A *lac* promoter was first chosen as target promoter. As expected, when the expression of both α-donor lacZα and α-acceptor lacZM15 were all under the control of the *lac* promoter, there was no significant difference of β-galactosidase activities between the lacZα reporter system and wild type lacZ reporter system. A moderately strong *ara* promoter was then chosen to be the detection promoter. Owing to its small size, the response of the lacZα-derived system was significantly more sensitive and higher than that of the wild type lacZ reporter system and commonly used mCherry reporter system. A weak *pbr* promoter was finally chosen to be the detection promoter. Due to the efficient expression profile of lacZα peptide, the response sensitivity of lacZα-derived system was demonstrated to be significantly higher than that of both wild type lacZ and mCherry-derived system. This study suggests that lacZα-derived reporter systems have numerous potential applications for monitoring low-level transcription in *E. coli*.

## Data Availability

The raw data supporting the conclusions of this manuscript will be made available by the authors, without undue reservation, to any qualified researcher.

## Author Contributions

C-YH designed the experimental protocol and drafted the manuscript. YG, H-QZ, and H-MW carried out the majority of the study. LL analyzed the data and corrected the manuscript. All authors read and approved the final manuscript.

## Conflict of Interest Statement

The authors declare that the research was conducted in the absence of any commercial or financial relationships that could be construed as a potential conflict of interest.
