# Supplementary material for: Improved Monitoring of Low-Level Transcription in Escherichia coli by a β-Galactosidase α-Complementation System
Source: Front Microbiol. 2019 Jun 26;10:1454. doi: 10.3389/fmicb.2019.01454 (PMC6607957; doi:10.3389/fmicb.2019.01454)
Supplement: Supplementary file 1 [file Data_Sheet_1.docx]

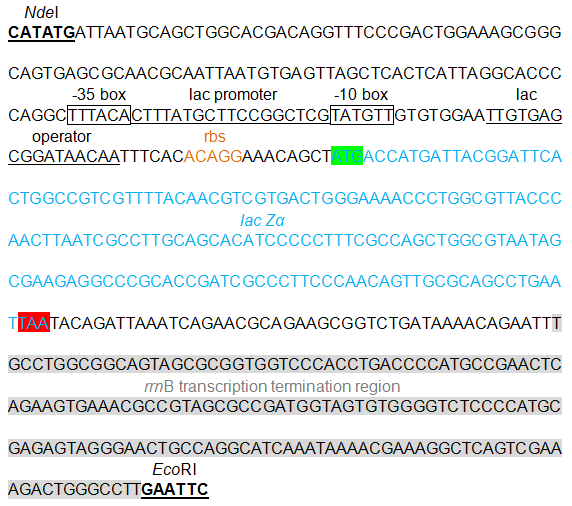


The cloning/expression region of pPlac-lacZα. *lacZα* is transcribed under the control of *lac* promoter.


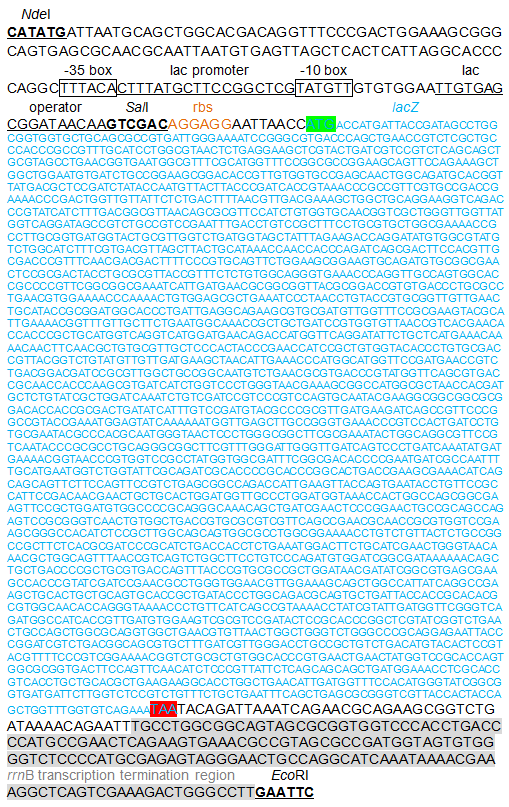


The cloning/expression region of pPlac-lacZ. *lacZ* is transcribed under the control of *lac* promoter.


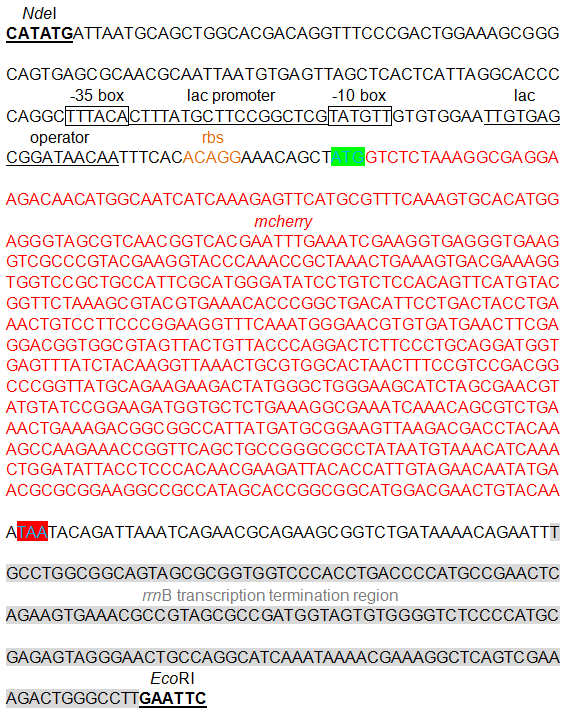


The cloning/expression region of pPlac-RFP. *mCherry* is transcribed under the control of *lac* promoter.


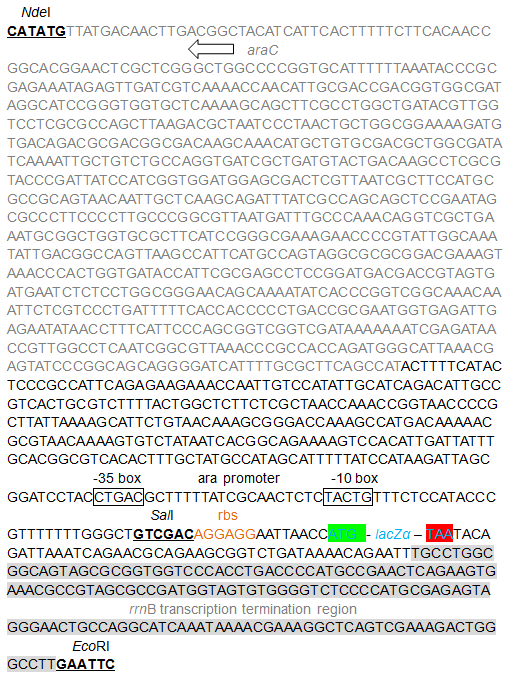


The cloning/expression region of pPara-lacZα. *lacZα* is transcribed under the control of *ara* promoter.


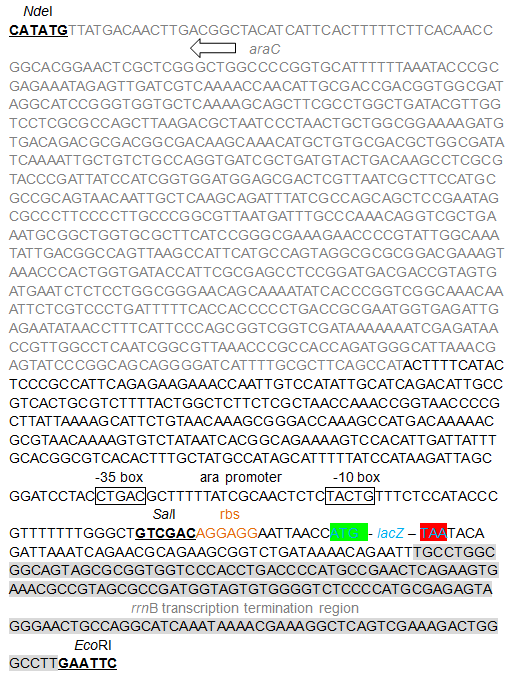


The cloning/expression region of pPara-lacZ. *lacZ* is transcribed under the control of *ara* promoter.


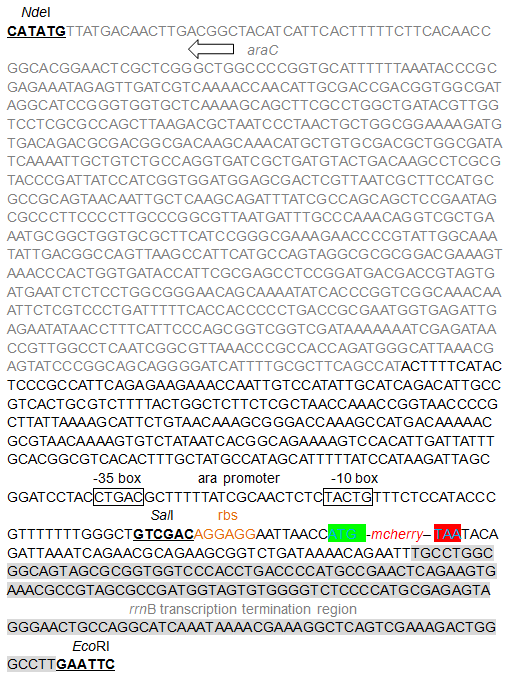


The cloning/expression region of pPara-RFP. *mCherry* is transcribed under the control of *ara* promoter.


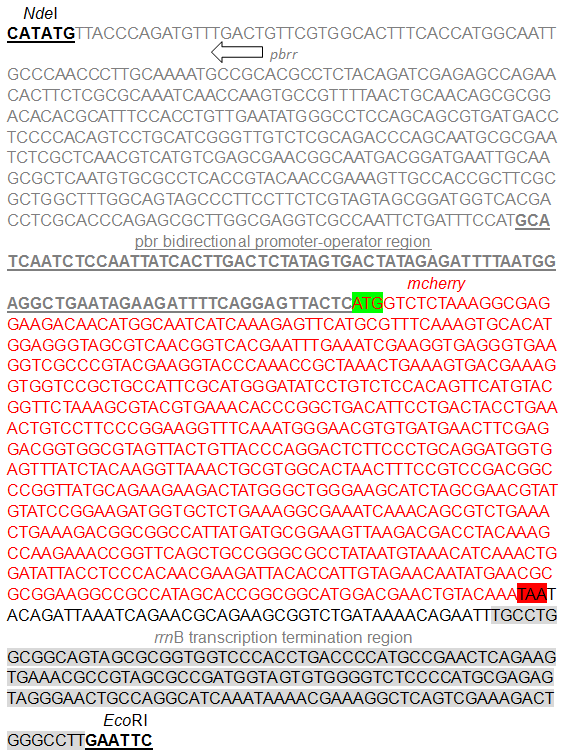


The cloning/expression region of pPpbr-RFP. The cassette including the *pbrR* gene and the divergent *pbr* promoter was inserted in front of *mCherry*.


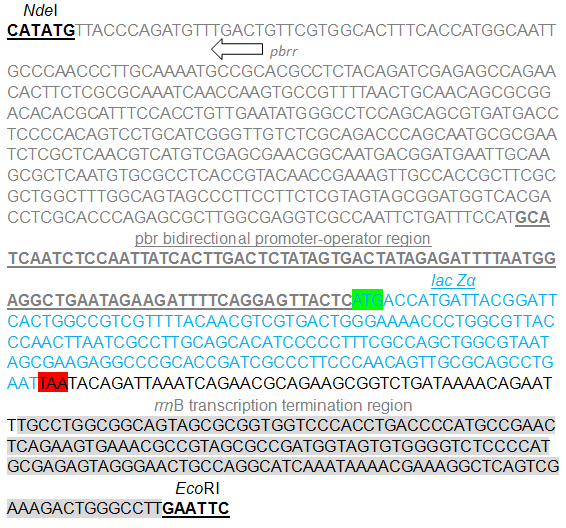


The cloning/expression region of pPpbr-lacZα. The cassette including the *pbrR* gene and the divergent *pbr* promoter was inserted in front of *lacZα*.


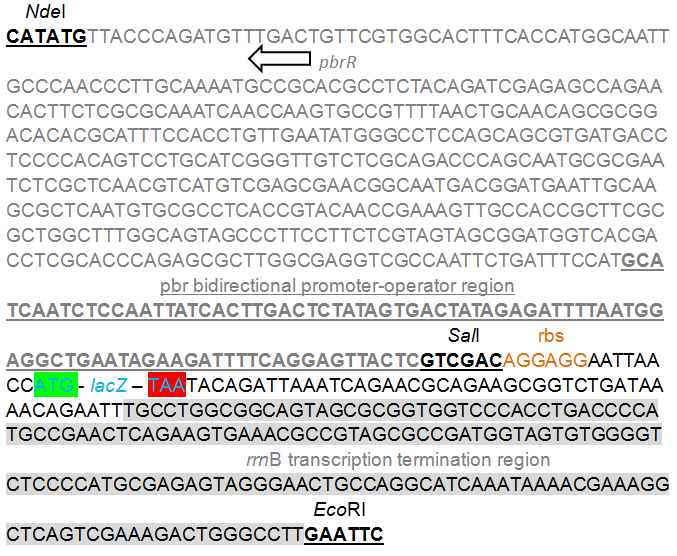


The cloning/expression region of pPpbr-lacZ. The cassette including the *pbrR* gene and the divergent *pbr* promoter was inserted in front of *lacZ*.

**Figure S1. The cloning/expression region of recombinant plasmids used in this study. DNA sequence and annotation data were all marked.**

**
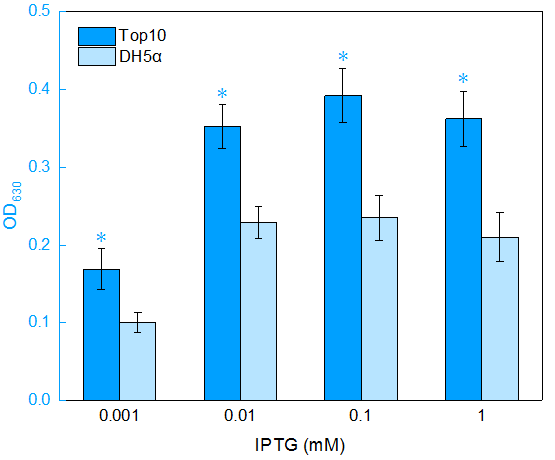
**

**Figure S2.** **Assay of the *lac* promoter activities in different hosts based on a lacZα reporter system.**

Overnight cultures were diluted in fresh 1% glucose-containing LB media. At the logarithmic growth phase, *E. coli* strains harboring pPlac-lacZα were exposed to different concentrations of IPTG. After a 4 h incubation at 37^o^C, the β-galactosidase activities were assayed. The optical density at 630 nm was normalized by dividing the OD­_630_ of the β-galactosidase activity determination system by the OD_600_ value of the induced culture. The data are representative of three independent experiments, and expressed as mean ± SEM. *A significant difference (*t* test, *P*<0.05) between *E. coli* Top10 and DH5α hosts.
